# BCL-2 family dysregulation in HTLV-1 and BLV pathogenesis and its implications for leukemogenesis and therapy

**DOI:** 10.1007/s11033-025-11216-5

**Published:** 2025-11-07

**Authors:** Mohammad Mehdi Akbarin, Zahra Farjami, Gabriel Eduardo Acevedo-Jiménez, Cecilia Rodríguez Murillo, Víctor David González-Fernández, Lucero de María Avila-De la Vega, Marcela Autran Martínez, Harim Jahaziel Nava Santos, Hugo Ramírez Álvarez

**Affiliations:** 1 Virology, Genetics, and Molecular Biology Laboratory, Faculty of Higher Studies Cuautitlan, Veterinary Medicine, National Autonomous University of Mexico, Campus 4, Cuautitlán Izcalli, Mexico; 2 Mashhad Medical Sciences-Medical School, Islamic Azad University, Mashhad, Iran; 3 Department of Biology, Damghan Branch, Islamic Azad University, Damghan, Iran; 4 FES-Cuautitlán, UNAM, Cuautitlán-Teoloyucan Highway, Km. 2.5, Cuautitlán Izcalli, Mexico; 5 San Sebastián Xhala, State of Mexico, Cuautitlán Izcalli, 54714 Mexico

**Keywords:** BCL-2 family, Apoptosis, Deltaretrovirus, HTLV-1, BLV, Tax protein, Adult t-cell Leukemia/Lymphoma (ATLL), Leukemogenesis, Viral persistence, Therapeutic targets

## Abstract

The B-cell Lymphoma 2 (BCL-2) family of proteins plays a fundamental role in maintaining the balance between cell survival and apoptosis, processes that are frequently manipulated by oncogenic viruses. This review explores the contribution of BCL-2 family members to the pathogenesis of deltaretroviruses, with emphasis on Human T-cell Leukemia Virus type 1 (HTLV-1) and Bovine Leukemia Virus (BLV). Both viruses employ viral oncoproteins such as Tax and HBZ (HTLV-1) or their BLV homologs to upregulate anti-apoptotic proteins, including BCL-2, BCL-xL (B-cell Lymphoma-extra Large), MCL-1 (Myeloid Cell Leukemia 1), and Bfl-1, while suppressing pro-apoptotic counterparts such as BCL-2–Associated X Protein ( BAX ), BIM (BCL-2–Interacting Mediator of Cell Death), and BID (BH3-Interacting Domain Death Agonist). This dysregulation prolongs the survival of infected lymphocytes and promotes clonal expansion, genomic instability, and malignant transformation. Importantly, studies demonstrate that BLV and HTLV-1 infection induce resistance to programmed cell death in lymphoid and even non-lymphoid cells, highlighting apoptosis evasion as a central mechanism of viral persistence. Therapeutically, BCL-2 family inhibition has shown promise in sensitizing transformed cells to apoptosis. Small-molecule inhibitors such as ABT-737 and Navitoclax, kinase inhibitors targeting NF-κB (Nuclear Factor kappa-light-chain-enhancer of Activated B Cells) and JAK/STAT (Janus Kinase/Signal Transducer and Activator of Transcription) pathways, and natural compounds including fucoxanthin, peridinin, and thymoquinone have demonstrated the ability to overcome apoptosis resistance in preclinical models. Recent strategies combining MCL-1 inhibitors with antiretroviral therapy or immune checkpoint blockade further highlight the translational potential of targeting BCL-2 pathways. Collectively, the evidence positions the BCL-2 family as a critical determinant of deltaretroviral persistence and leukemogenesis, and as a promising therapeutic axis for the development of novel treatments for HTLV-1–Associated Myelopathy/Tropical Spastic Paraparesis (HAM/TSP) and BLV-associated leukosis.

## Introduction

The B-cell Lymphoma 2 (BCL-2) family of proteins plays a pivotal role in regulating the balance between cell survival and programmed cell death [1]. Members of this family are defined by the presence of one or more conserved BCL-2 homology (BH) domains, which mediate protein–protein interactions critical for controlling Mitochondrial Outer Membrane Permeabilization (MOMP). The functional classification of these proteins provides further insight: specifically, members are divided into anti-apoptotic proteins (such as BCL-2, BCL-xL (B-cell Lymphoma-extra Large), and MCL-1 Myeloid Cell Leukemia 1) and pro-apoptotic members. The latter group is further subdivided into multidomain effectors (BAX [BCL-2–Associated X Protein], BAK [BCL-2 Homologous Antagonist/Killer]) and BH3-only proteins (BIM [BCL-2–Interacting Mediator of Cell Death], BID (BH3-Interacting Domain Death Agonist), PUMA [p53-Upregulated Modulator of Apoptosis]) [2, 3].

**Fig. 1:**
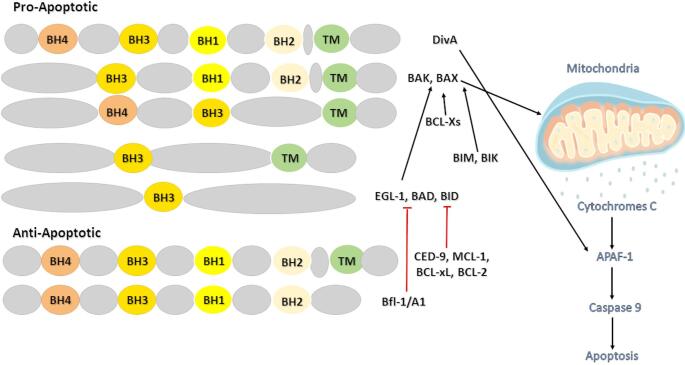
The BCL-2 family of proteins and their role in the regulation of apoptosis. The family is divided into anti-apoptotic and pro-apoptotic members based on their structure and function. Anti-apoptotic proteins, including BCL-2 (B-cell Lymphoma 2), BCL-xL (B-cell lymphoma-extra-large), MCL-1 (Myeloid cell leukemia sequence 1), and Bfl-1/A1 (BCL2-related protein A1), contain multiple BCL-2 homology (BH) domains (BH1-BH4) and a transmembrane (TM) domain, enabling them to stabilize mitochondrial membranes and inhibit apoptosis. Pro-apoptotic members are subdivided into multidomain proteins such as BAX (BCL-2–Associated X Protein and BAK (BCL-2 Homologous Antagonist/Killer, which contain BH1–BH3 domains and TM domains, and BH3-only proteins including BIM (BCL2-like 11), BID (BH3-Interacting Domain Death Agonist), PUMA (p53 upregulated modulator of apoptosis), BAD (BCL-2–Associated Agonist of Cell Death), BIK (BCL2-interacting killer), and EGL-1 (programmed cell death activator homolog), which act as sentinels of cellular stress. These BH3-only proteins antagonize anti-apoptotic proteins and activate BAX and BAK. Upon activation, BAX and BAK oligomerize in the mitochondrial outer membrane, leading to mitochondrial outer membrane permeabilization (MOMP) and the release of cytochrome c into the cytoplasm. Released cytochrome c interacts with APAF-1 (apoptotic protease activating factor-1), triggering the activation of caspase-9 and initiating the caspase cascade that results in programmed cell death. This balance between anti- and pro-apoptotic BCL-2 family proteins is central to cell fate determination, and its dysregulation plays a crucial role in oncogenesis and viral pathogenesis. *All figures were created using Microsoft PowerPoint 365*

Based on structure and function, the BCL-2 family is divided into three subgroups: anti-apoptotic proteins, pro-apoptotic effectors, and pro-apoptotic BH3-only proteins. First, the anti-apoptotic subgroup, including BCL-2, BCL-xL, and MCL-1, typically contains four BH domains (BH1-BH4) and preserves cell survival by sequestering pro-apoptotic proteins. Next, the pro-apoptotic effectors, such as BAX and BAK, possess three BH domains and are responsible for directly inducing MOMP and cytochrome c release, leading to caspase activation. In contrast, the BH3-only proteins (such as BIM, BID, and PUMA) contain a single BH3 domain and act as sentinels of cellular stress, promoting apoptosis by neutralizing anti-apoptotic proteins or directly activating BAX and BAK. This intricate balance between survival and death signals, governed by the structural interplay of BCL-2 related proteins, is critical for cellular homeostasis and is frequently hijacked during viral infections and tumorigenesis [2, 3] (Fig. 1). The equilibrium maintained by the BCL-2 family is essential for tissue homeostasis, immune system regulation, and cellular responses to stress. Dysregulation of this system often leads to uncontrolled cell proliferation or premature cell death, outcomes that are closely linked to cancer development, viral persistence, and immune evasion.

### BCL-2 family and viral oncogenesis

Human T-cell Leukemia Virus type 1 (HTLV-1) and Bovine Leukemia Virus (BLV) are closely related members of the Deltaretrovirus genus that illustrate the pathological importance of apoptosis regulation. HTLV-1 is the causative agent of Adult T-cell Leukemia/Lymphoma (ATLL), a highly aggressive and fatal malignancy, and is also linked to chronic inflammatory disorders such as HTLV-1–Associated Myelopathy/Tropical Spastic Paraparesis (HAM/TSP) [4, 5]. BLV, the animal counterpart, causes enzootic bovine leukosis, a widespread hematological malignancy in cattle, leading to significant economic losses in the dairy and beef industries [6, 7]. Although interspecies differences exist, both viruses share molecular strategies to subvert host apoptosis, thereby allowing viral persistence and malignant transformation. Notably, while HTLV-1 accounts for thousands of deaths annually, BLV infection continues to spread worldwide, with zoonotic implications still under investigation [6, 7].

Apoptosis, or programmed cell death, is a central process in the host defense against viral infections. By eliminating infected or damaged cells, apoptosis limits viral spread and prevents oncogenic transformation. However, deltaretroviruses exploit the host apoptotic machinery for their benefit [8, 9]. Viral regulatory proteins, such as Tax and HBZ in HTLV-1 and their BLV homologs, modulate cellular survival pathways by influencing BCL-2 family expression and function [10]. Increased expression of anti-apoptotic BCL-2 proteins has been reported in HTLV-1–infected T-cells and BLV-infected B-cells, contributing to the prolonged survival of transformed lymphocytes [11]. This dysregulation not only facilitates viral persistence but also promotes oncogenesis by preventing the clearance of genetically unstable cells. Consequently, resistance to apoptosis is a hallmark of HTLV-1 and BLV pathogenesis and a key factor in the poor prognosis and high mortality rates observed in ATLL and bovine leukosis.

Despite advances in understanding the oncogenic role of deltaretroviruses, there remains a significant gap in knowledge about the precise contribution of the BCL-2 family to viral persistence, immune evasion, and leukemogenesis. While studies have demonstrated altered expression patterns of anti- and pro-apoptotic proteins in infected cells, the mechanisms by which BCL-2 family regulation supports deltaretroviral survival and malignant transformation remain insufficiently characterized. In particular, little is known about how different members of the family interact with viral proteins and cellular signaling pathways to dictate the balance between cell death and survival.

### Search strategy

To ensure comprehensive coverage of the available literature, we conducted systematic searches in PubMed and Web of Science databases for studies published up to August 2025. The search combined controlled vocabulary and free-text terms related to deltaretrovirus infection and apoptosis, including: “HTLV-1,” “HTLV-2,” “BLV,” “Adult T-cell Leukemia/Lymphoma (ATLL),” “HTLV-1-Associated Myelopathy/Tropical Spastic Paraparesis (HAM/TSP),” “BCL-2 family,” “BCL-like proteins,” “apoptosis,” and “cell survival.”

Additional references were identified by examining the bibliographies of key experimental and review papers. Only peer-reviewed studies published in English that investigated the molecular mechanisms or therapeutic modulation of BCL-2 family proteins in the context of HTLV-1 or BLV infection were included. This narrative synthesis focuses on findings relevant to viral modulation of apoptosis and therapeutic targeting of BCL-2-regulated pathways.

This review aims to synthesize and critically analyze current evidence regarding the role of BCL-2 family proteins in deltaretrovirus infection, with particular focus on HTLV-1 and BLV. By integrating findings on the expression, regulation, and functional implications of pro- and anti-apoptotic BCL-2 members, this review seeks to elucidate their contribution to viral persistence, immune evasion, and leukemogenesis. Moreover, it highlights emerging therapeutic strategies targeting BCL-2 pathways as promising avenues for controlling HTLV-1–related malignancies and BLV-associated leukosis.

## Dual role of BLV in apoptosis induction and Inhibition

The BCL-2 family of proteins plays a critical role in the pathogenesis of BLV infection by regulating the delicate balance between cell survival and apoptosis. This process directly influences viral persistence and leukemogenesis. BLV, a deltaretrovirus closely related to HTLV-1, manipulates host apoptotic pathways during infection to favor the survival of infected cells, mainly through the upregulation of anti-apoptotic BCL-2 affiliated proteins such as BCL-2 and MCL-1, which protect infected lymphocytes from programmed cell death [12]. At the same time, pro-apoptotic proteins, including BAX, BAK, and BH3-only members, are suppressed or functionally inhibited, preventing the clearance of virally transformed or genetically unstable cells. This disruption of the apoptotic equilibrium not only ensures long-term viral persistence within the host but also contributes to genomic instability and malignant transformation.

Szynal, M. et al. investigated how the BLV-Tax oncoprotein alters B-cell homeostasis and contributes to leukemogenesis [13]. The authors demonstrated that Tax expression disrupts the standard control of B-cell survival by inducing constitutive activation of the Nuclear Factor kappa-light-chain-enhancer of Activated B Cells (NF-κB) signaling pathway, which in turn leads to upregulation of the anti-apoptotic protein BCL-2 [13]. This Tax-mediated signaling promotes prolonged survival of BLV-infected B-cells by protecting them from apoptosis, thereby allowing clonal expansion and accumulation of genetically unstable cells that favor malignant transformation. Notably, the study highlights a mechanistic link between Tax-driven NF-κB activation and BCL-2 overexpression, identifying a critical pathway by which BLV manipulates host apoptosis to establish persistent infection and drive oncogenesis [13]. The results suggest that targeting BCL-2 or NF-κB signaling could represent a therapeutic approach to control BLV-associated leukosis.

In addition, in 2005, Takahashi, M. et al. explored the dual role of bovine leukemia virus (BLV) in modulating apoptosis, showing that the virus can both induce and inhibit programmed cell death depending on the stage of infection and the cellular context [14]. Early in infection, BLV may trigger apoptosis as part of the host defense response. However, in persistently infected and transformed B-cells, the virus actively blocks apoptosis to ensure long-term survival and proliferation. A key finding is the involvement of the BCL-2 family in this process: BLV infection is associated with increased expression of anti-apoptotic proteins such as BCL-2 and MCL-1, which protect infected lymphocytes from death, while pro-apoptotic members, including BAX and BIM, are downregulated or functionally inhibited [14]. This imbalance favors cell survival, viral persistence, and accumulation of oncogenic mutations, ultimately contributing to the development of enzootic bovine leukosis. These results highlight the BCL-2 family as a central regulator of the interplay between apoptosis induction and inhibition in BLV infection, underlining its importance in viral pathogenesis and leukemogenesis.

Erskine, R. J. et al. in 2011, examined how bovine leukemia virus (BLV) infection alters lymphocyte proliferation and apoptosis in dairy cattle, providing insights into viral persistence and disease progression [15]. The results showed that BLV-infected animals exhibit increased lymphocyte proliferation, particularly in B-cells, accompanied by a significant reduction in apoptosis. This resistance to programmed cell death was strongly associated with dysregulation of the BCL-2 family, as infected lymphocytes demonstrated elevated expression of anti-apoptotic proteins such as BCL-2. At the same time, pro-apoptotic members like BAX were reduced or functionally suppressed [15]. The imbalance between survival and death signals created an environment that favored the expansion and accumulation of infected cells, promoting viral persistence and predisposing to leukemogenesis. These findings emphasize the critical role of the BCL-2 family in modulating apoptosis during BLV infection and underscore its contribution to the pathogenesis of enzootic bovine leukosis.

Furthermore, in 2019, Martinez Cuesta, L. et al. investigated the impact of bovine leukemia virus (BLV) infection on bovine mammary epithelial cells, focusing on cellular survival, proliferation, and apoptosis [16]. The findings revealed that BLV can infect and alter the behavior of mammary epithelial cells, leading to enhanced cell survival and resistance to apoptosis. A key mechanism underlying this effect was the modulation of the BCL-2 family of proteins, as infected cells showed increased expression of the anti-apoptotic protein BCL-2 and decreased levels or activity of pro-apoptotic proteins such as BAX [16]. This imbalance protected mammary epithelial cells from programmed cell death and promoted their abnormal proliferation, creating a microenvironment favorable for viral persistence and potentially contributing to oncogenic transformation. These results underscore the role of BCL-2 family dysregulation in non-lymphoid cells during BLV infection and suggest that viral interference with apoptotic pathways extends beyond lymphocytes, broadening the scope of BLV-associated pathogenesis.

In 2024, Ladera Gomez, M. E. et al. investigated the mechanisms underlying apoptosis in milk and blood cells from bovines infected with Bovine Leukemia Virus (BLV), categorized by high proviral load (HPL) and low proviral load (LPL) [17]. The research focuses on the expression of tumor necrosis factor-alpha (TNF-α), TNF receptors (TNF-RI and TNF-RII), anti-apoptotic BCL-2, and pro-apoptotic BAX proteins in somatic milk cells (SC) and peripheral blood mononuclear cells (PBMCs) using quantitative reverse transcription-polymerase chain reaction (RT-qPCR). The study found that HPL animals exhibited a significant decrease in TNF-α expression in SC compared to non-infected bovines. In contrast, TNF-RI expression was significantly higher in PBMCs from HPL animals compared to LPL animals. Additionally, both BCL-2 and BAX mRNA levels were significantly increased in SC from LPL animals compared to non-infected bovines, with the BCL-2/BAX ratio indicating an anti-apoptotic profile in both LPL and HPL animals [17]. In PBMCs, reduced BAX expression was observed in HPL animals compared to LPL subjects, suggesting that the increased BCL-2 expression may contribute to negative apoptosis regulation in the mammary gland induced by BLV infection [17]. These findings provide new insights into the mechanisms of mammary cell death in BLV-infected bovines during lactation.

Moreover, Mukantayev, K. et al., this year, explored the potential of combining recombinant bovine interleukin-15 (rbIL15) with monoclonal antibodies against CTLA-4 and PD-L1 to enhance immune responses in BLV-infected cattle [18]. The research demonstrates that rbIL15 induces the expression of the anti-apoptotic protein BCL-2 and transcription factors STAT3 (Signal Transducer and Activator of Transcription 3) and STAT5 (Signal Transducer and Activator of Transcription 5) in peripheral blood mononuclear cells (PBMCs) from both healthy and BLV-infected cattle. Additionally, the combination treatment significantly increases interferon-gamma (IFN-γ) production in PBMCs from BLV-infected cattle, suggesting improved immune activation [18]. These findings indicate that blocking CTLA-4 and PD-L1 enhances the efficacy of rbIL15 immunotherapy, potentially offering a novel approach to modulate apoptosis and proliferation pathways in BLV-infected bovines.

In conclusion, the collective evidence underscores the pivotal role of the BCL-2 family in regulating apoptosis during BLV infection, shaping both viral persistence and disease progression. Across multiple studies, BLV has been shown to manipulate host cell survival by upregulating anti-apoptotic proteins such as BCL-2 and MCL-1 while suppressing pro-apoptotic members like BAX and BIM, thereby promoting the survival and clonal expansion of infected B-cells and, in some cases, mammary epithelial cells. This dysregulation, often mediated by viral proteins such as Tax and by altered signaling through pathways like NF-κB, creates an environment conducive to genomic instability and leukemogenesis. Furthermore, recent immunotherapeutic approaches combining rbIL15 with CTLA-4 and PD-L1 blockade demonstrate that modulating BCL-2-mediated survival pathways can enhance immune responses and restore apoptotic control. These highlights both the mechanistic significance of BCL-2 family proteins in BLV pathogenesis and their potential as therapeutic targets to limit viral persistence and malignant transformation.

## BCL-2 function in HTLV infection

**Fig. 2:**
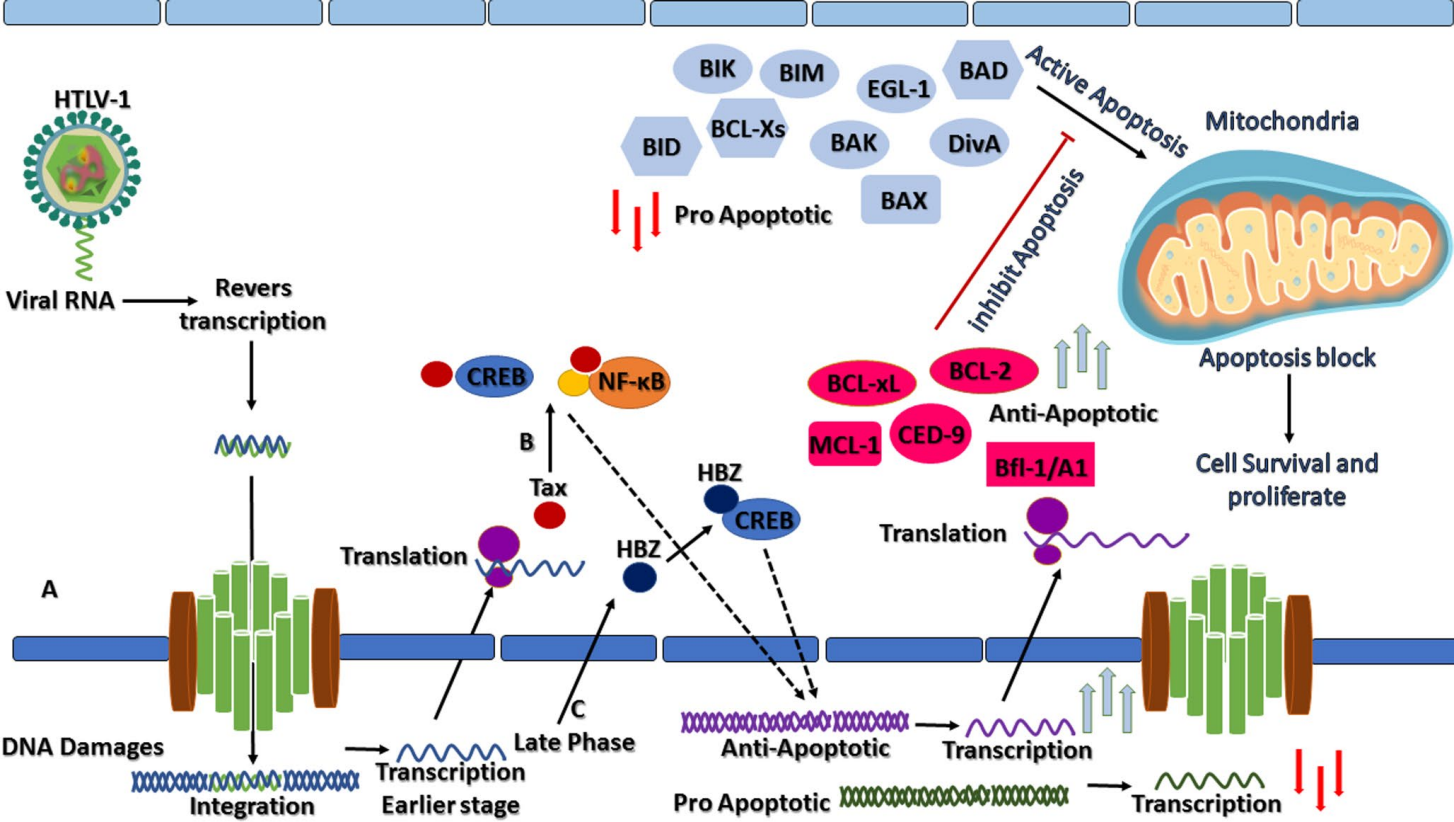
The schematic illustrates the cooperative actions of Human T-cell Leukemia Virus type 1 (HTLV-1) viral proteins Tax and HTLV-1 basic leucine zipper factor (HBZ) in manipulating host apoptotic pathways through regulation of the B-cell lymphoma 2 (BCL-2) family of proteins. **A** viral entry and integration of the viral genome, expression of Tax and HBZ acts in a temporally and functionally complementary manner to promote cell survival. **B** Tax initiates early activation of pro-survival signaling cascades, including Nuclear Factor kappa-light-chain-enhancer of Activated B Cells (NF-κB) and cAMP Response Element–Binding Protein (CREB), leading to transcriptional upregulation of BCL-2 anti-apoptotic members, including: BCL-2, BCL-xL, MCL-1, and Bfl-1/A1. **C**: HBZ subsequently sustains and stabilizes this anti-apoptotic state by enhancing BCL-2 and MCL-1 expression and repressing BH3-only pro-apoptotic factors such as BIM, BID (BH3-Interacting Domain Death Agonist), and PUMA through both transcriptional and protein–protein interaction mechanisms. Together, Tax-driven activation and HBZ-mediated maintenance converge to suppress pro-apoptotic proteins (BAX, BAK, BIM, BID, BAD, and DivA), thereby preventing Mitochondrial Outer Membrane Permeabilization (MOMP), cytochrome c release, and caspase activation. This cooperative blockade of apoptosis supports persistent survival of infected T lymphocytes, resistance to DNA damage-induced cell death, and contributes to viral persistence and leukemogenesis. *All figures were created using Microsoft PowerPoint 365*

The BCL-2 family of proteins plays a central role in regulating apoptosis, maintaining the delicate balance between cell survival and programmed cell death, and thereby influencing cellular homeostasis and immune responses. In the context of HTLV-1 infection, this family of proteins is particularly important because the virus targets CD4⁺ T-cells and manipulates apoptotic pathways to promote the survival of infected cells, a critical step for viral persistence and the development of ATLL. HTLV-1 encodes viral oncogenes, such as Tax and HBZ, which can disrupt normal apoptotic regulation by modulating the expression and activity of anti-apoptotic BCL-2 related proteins, while suppressing pro-apoptotic proteins, thus allowing infected cells to evade programmed cell death [10, 19] (Fig. 2). This interference not only facilitates clonal expansion of infected T-cells but also contributes to genomic instability and malignant transformation, positioning the BCL-2 memeber as a key player in HTLV pathogenesis and a potential target for therapeutic intervention. Therefore, the interaction between HTLV and the BCL-2 family can be considered in the context of higher expression of anti-apoptotic BCL-2 family, a decrease in pro-apoptotic members, and the induction of genetic variation in death signals, which in turn changes related signaling pathways such as NF-kappa B proteins.

### Alteration in BCL-2 expression

The alteration in BCL-2 family expression has been investigated in several studies involving human and mouse models of HTLV infection. Most of these studies reported a significant increase in BCL-2 anti-apoptotic protein expression [20–22]. This condition is not limited only to the HTLV-1-associated disease, while in some other types of autoimmune disorders, such as Sjogren’s syndrome, indirect interaction of Tax with other pathways, such as CD40/CD40 ligand and X chromosome-linked inhibitor of apoptosis protein (XIAP) proteins [20, 23].

Nicot, C. et al., primarily investigated the effects of human T-cell lymphotropic virus type I (HTLV-I) on the regulation of apoptosis in human endothelial cells [24]. The findings demonstrated that chronic expression of HTLV-I in endothelial cells leads to a significant upregulation of the anti-apoptotic protein BCL-2 [24]. This activation of BCL-2 expression enhanced cell survival and resistance to apoptosis, suggesting a key role for the BCL-2 family in maintaining the persistence of HTLV-I–infected cells. By promoting cell survival, HTLV-I–induced BCL-2 activation may contribute to viral persistence and play a role in the pathogenesis of HTLV-associated diseases, particularly in the development of oncogenic processes where evasion of apoptosis is critical.

In addition, Sakai, T. et al. compared serum levels of apoptosis-regulating molecules in patients with multiple sclerosis (MS) and HTLV-I-associated myelopathy/tropical spastic paraparesis (HAM/TSP) [25]. The results revealed distinct alterations in the expression of BCL-2 family proteins, particularly highlighting increased levels of the anti-apoptotic molecule BCL-2 in HAM/TSP patients. This upregulation suggests that HTLV-I infection promotes cell survival by suppressing apoptosis, thereby contributing to the persistence of infected cells and the chronic inflammatory state characteristic of HAM/TSP [25]. These findings underscore the role of the BCL-2 family in HTLV-I pathogenesis and point to dysregulated apoptotic pathways as a potential mechanism linking viral infection to neuroinflammatory disease progression.

Moreover, the regulation of anti-apoptotic pathways in the context of HTLV infection and ATLL was examined by Nicot, C. et al. The findings showed that both HTLV-I and HTLV-II strongly upregulate the expression of the BCL-2 family member BCL-xL in infected T cells, both in vitro and in ex vivo ATLL patient samples [26]. Elevated BCL-xL levels conferred resistance to apoptosis, thereby enhancing the survival of HTLV-infected cells and supporting viral persistence. In ATLL, this anti-apoptotic effect may facilitate malignant transformation by protecting transformed T cells from programmed cell death. These results highlight the central role of BCL-xL in HTLV pathogenesis and suggest that targeting BCL-2 family proteins could represent a therapeutic strategy for HTLV-associated malignancies.

Moreover, Macaire, H. et al. in 2012, demonstrated that the HTLV-1 viral protein Tax plays a pivotal role in promoting the survival of infected T cells by inducing the expression of Bfl-1, an anti-apoptotic member of the BCL-2 family [27]. Tax-mediated upregulation of Bfl-1 enhanced resistance to apoptosis in HTLV-1–infected T cells, thereby supporting viral persistence and contributing to leukemogenesis [27]. These findings highlight Bfl-1 as a key survival factor in HTLV-1 infection, showing that manipulation of BCL-2 family proteins is a critical mechanism through which HTLV-1 evades cell death and promotes the development of ATLL.

Furthermore, Choi, Y. B. et al. revealed that the HTLV-1 Tax protein enhances the survival and oncogenic potential of infected T cells by targeting the anti-apoptotic BCL-2 family member MCL-1 [28]. Tax was shown to stabilize MCL-1 through TRAF6-dependent K63-linked polyubiquitination, preventing its degradation and thereby maintaining high levels of this survival protein [28]. The accumulation of stabilized MCL-1 promoted resistance to apoptosis, sustained cell survival, and facilitated cellular transformation, key events in the pathogenesis of ATLL. These findings emphasize the critical role of BCL-2 family regulation in HTLV-1–driven oncogenesis and identify MCL-1 stabilization as a central mechanism by which Tax contributes to leukemic progression.

In 2024, Fajami, Z. et al. evaluated the expression of the anti-apoptotic BCL-2 family member BCL-xL alongside the viral genes Tax and HBZ in patients with ATLL [10]. The results showed that BCL-xL expression was significantly elevated in ATLL samples, consistent with its role in promoting cell survival and resistance to apoptosis [10]. Significantly, the increased expression of BCL-xL was associated with the presence of viral oncogenes Tax and HBZ, suggesting that both viral factors contribute to the dysregulation of apoptotic pathways. These findings reinforce the concept that HTLV-1 manipulates BCL-2 family proteins, particularly BCL-xL, to sustain the survival of malignant T cells and support leukemogenesis.

HTLV can also decrease the expression of pro-apoptotic genes in infected cells. Krueger, A. et al. investigated how HTLV-1 Tax modulates apoptotic signaling to enhance the survival of infected T cells [29]. The results demonstrated that Tax protects cells from CD95 (Fas)-mediated apoptosis by upregulating the expression of c-FLIP, a key inhibitor of caspase-8 activation in the death receptor pathway. Although c-FLIP is not a member of the BCL-2 family, its interaction with apoptotic signaling complements the anti-apoptotic effects mediated by BCL-2 family proteins. By simultaneously activating BCL-2 members (such as BCL-2, BCL-xL, Bfl-1, and MCL-1) and death receptor inhibitors like c-FLIP, HTLV-1 creates a robust survival network that allows infected T cells to resist apoptosis, persist long-term, and undergo leukemic transformation [29].

In addition, in 2014, Muhleisen et al. showed that the HTLV-1 oncoprotein Tax enhances the survival of infected T cells by interfering with the pro-apoptotic arm of the BCL-2 family [30]. Specifically, Tax suppressed the expression of the BH3-only proteins BID and BIM, both of which are critical initiators of the mitochondrial apoptosis pathway [30]. By downregulating these pro-apoptotic factors, Tax tipped the balance of BCL-2 family activity toward cell survival, thereby protecting HTLV-1–infected T cells from apoptosis. This suppression of BID and BIM complements the Tax-driven upregulation and stabilization of anti-apoptotic BCL-2 family (such as BCL-xL, Bfl-1, and MCL-1), collectively ensuring persistence of infected cells and contributing to leukemogenesis in ATLL.

### DNA damage and BCL-2 mediated survival

A critical component of the cellular response to DNA damage is the regulation of apoptosis, primarily governed by the BCL-2 family of proteins [31]. HTLV-1 infection disrupts this delicate balance through the action of its viral proteins, particularly Tax and HBZ, which not only drive DNA damage through aberrant proliferation and oxidative stress but also activate BCL-2 anti-apoptotic members while suppressing pro-apoptotic counterparts [32, 33]. This dual effect enables infected cells to evade apoptosis despite accumulating genomic lesions, thereby promoting clonal expansion and malignant transformation. Understanding how HTLV-1 exploits the BCL-2 family in the context of DNA damage offers key insights into viral persistence, leukemogenesis, and potential therapeutic targets for ATLL.

Maruyama, K. et al. explored how HTLV-1 infection drives the transformation of human lymphocytes through the induction of genetic instability. The results showed that HTLV-1–infected cells accumulate chromosomal abnormalities and DNA damage, which contribute to malignant progression [34]. Importantly, despite ongoing genomic injury, infected lymphocytes resist apoptosis due to the dysregulation of apoptotic pathways, including the activation of anti-apoptotic BCL-2 family proteins [34]. By shifting the balance toward cell survival, HTLV-1 enables damaged and genetically unstable lymphocytes to persist and clonally expand, thereby establishing a foundation for leukemogenesis. These findings underscore the cooperative role of HTLV-1–induced genomic instability and BCL-2 family–mediated apoptosis resistance in the development of ATLL.

In addition, Kao, S. Y. et al. investigated the effects of HTLV-1 Tax on apoptosis in response to UV-induced DNA damage. The results demonstrated that, under certain conditions, Tax can sensitize cells to apoptosis independently of p53, highlighting a context-dependent role of Tax in cell death regulation [35]. While Tax is generally associated with the upregulation of BCL-2, BCL-xL, Bfl-1, and MCL-1 to promote survival, this study showed that Tax can also trigger apoptotic pathways in the presence of severe DNA damage, indicating a complex interplay between viral protein activity and cellular stress responses [35]. Overall, the findings suggest that although HTLV-1 commonly exploits the BCL-2 family to protect infected cells, Tax retains the capacity to modulate apoptosis dynamically depending on the cellular context and genotoxic stress.

In 2001, Tsukasaki, K. et al. examined the association between TNF-α gene polymorphisms and susceptibility to ATLL in HTLV-1 carriers. The results indicated that specific TNF-α promoter variants correlated with a higher risk of ATLL development, likely by influencing inflammatory and apoptotic signaling pathways [36]. In the context of HTLV-1 infection, these polymorphisms may exacerbate the virus-driven dysregulation of apoptosis, including the upregulation of anti-apoptotic BCL-2 affiliated proteins such as BCL-2, BCL-xL, and MCL-1, which protect infected T cells from programmed cell death [36]. By combining viral manipulation of BCL-2 family proteins with host genetic predisposition affecting apoptosis, HTLV-1–infected individuals carrying susceptible TNF-α variants experience enhanced cell survival, increased clonal expansion, and greater risk of leukemic transformation.

Moreover, in 2011, Abou-Kandil, A. et al. investigated how DNA damage influences HTLV-1 transcription through the viral long terminal repeat (LTR) and the involvement of apoptotic regulators. The results demonstrated that DNA-damaging agents activate caspase-9, which in turn enhances HTLV-1 L-driven gene expression [37]. Although caspase-9 is typically associated with the intrinsic apoptosis pathway, HTLV-1–infected cells often resist apoptosis due to upregulation of anti-apoptotic BCL-2 family proteins such as BCL-2, BCL-xL, and MCL-1 [37]. This interplay allows the virus to exploit DNA damage signaling to promote viral transcription while maintaining cell survival, illustrating how HTLV-1 manipulates both apoptotic and transcriptional pathways to sustain infection and facilitate leukemogenesis.

Chaib-Mezrag, H. et al. in 2014, demonstrated that the HTLV-1 oncoprotein Tax disrupts DNA replication by slowing or stalling replication forks, leading to increased DNA breaks in specific genomic regions associated with oncogenes [38]. Although Tax induces genomic instability, HTLV-1–infected cells often evade apoptosis due to the upregulation of anti-apoptotic BCL-2 family proteins, including BCL-2, BCL-xL, Bfl-1, and MCL-1 [38]. This coordinated effect allows cells harboring DNA damage to survive and proliferate, facilitating clonal expansion and increasing the risk of malignant transformation. The findings highlight how Tax couples its DNA damage induction with BCL-2 family–mediated apoptosis resistance, promoting both viral persistence and the development of ATLL.

### Interactions with oncogenes and host factors

Multiple studies have shown that Tax upregulates BCL-xL and BCL-2 via activation of NF-κB and CREB (cAMP Response Element–Binding Protein) signaling pathways, enhancing resistance to programmed cell death and supporting viral persistence and leukemogenesis. While Tax predominantly promotes cell survival, it can paradoxically induce apoptosis in distinct contexts through interactions with co-activators like p300, emphasizing its context-dependent dual function. Other cellular factors, including p21^WAF1 (cyclin-dependent kinase inhibitor) and proto-oncogenes such as c-Myc (Cellular Myelocytomatosis Oncogene), further cooperate with Tax to reinforce anti-apoptotic signaling.

Tsukahara, T. et al. demonstrated that the HTLV-1 oncoprotein Tax promotes resistance to apoptosis in infected T cells by inducing the expression of the anti-apoptotic BCL-2 family member BCL-xL [39]. The findings showed that Tax activates NF-κB signaling, which directly upregulates BCL-xL expression in T-cell transfectants, thereby enhancing their survival despite apoptotic stimuli [39]. This mechanism underscores how HTLV-1 manipulates host survival pathways, with Tax serving as a central regulator that links viral oncogenic signaling to BCL-2 family activation. The upregulation of BCL-xL through NF-κB not only protects HTLV-1–infected cells from programmed cell death but also contributes to their persistence and the development of ATLL.

Moreover, Nicot, C. et al. explored how the HTLV-1 Tax protein, known for promoting cell survival, can paradoxically induce apoptosis under certain conditions [40]. Their findings showed that Tax interacts with the transcriptional co-activator p300 to trigger distinct signaling pathways that activate caspases and promote apoptosis [40]. In contrast, in other contexts, Tax upregulates anti-apoptotic BCL-2 family proteins (such as BCL-2, BCL-xL, Bfl-1, and MCL-1), supporting cell survival. Thus, Tax’s dual role transitions based on cellular environment and stress signals: it can either protect infected T cells from apoptosis by manipulating BCL-2 family proteins or promote caspase-dependent apoptosis under specific conditions. This dynamic contributes to the complexity of HTLV-1 pathogenesis and may facilitate viral replication, immune evasion, or clonal selection during leukemogenesis.

Mori, N. et al. demonstrated that the HTLV-1 Tax protein enhances the survival of infected T cells by directly upregulating the anti-apoptotic BCL-2 family member BCL-xL. The results showed that Tax induces BCL-xL expression through activation of both NF-κB and CREB signaling pathways, thereby providing dual transcriptional control to strengthen apoptosis resistance [41]. Elevated BCL-xL levels allowed HTLV-1–infected T cells to evade programmed cell death and persist despite apoptotic signals, supporting long-term viral survival and contributing to leukemogenesis. These findings reinforce the central role of BCL-2 family manipulation in HTLV-1 pathogenesis and identify NF-κB and CREB-mediated induction of BCL-xL as a key mechanism by which Tax promotes oncogenic transformation.

In 2005, Akita, K. et al. investigated the role of p21^WAF1 in apoptosis regulation within the context of HTLV-1 Tax expression. The results showed that p21^WAF1 enhances NF-κB signaling in Tax-expressing rat fibroblasts, leading to the upregulation of the anti-apoptotic BCL-2 protein [42]. By promoting BCL-2 expression, p21^WAF1 contributed to apoptosis resistance and prolonged cell survival, thereby cooperating with Tax in creating a pro-oncogenic environment [42]. These findings highlight an indirect but important mechanism through which HTLV-1 manipulates host signaling networks, linking p21^WAF1 activity to NF-κB–mediated induction of BCL-2, and ultimately reinforcing the anti-apoptotic phenotype of Tax-expressing cells.

In addition to the NF-κB signaling pathway, studies have demonstrated that proto-oncogene proteins, such as c-Myc, may also, via HTLV infection, induce the inhibition of apoptosis [43, 44].

Bellon, M. et al. in 2024, demonstrated that the HTLV-1 oncoprotein Tax promotes oncogenic transformation by inactivating the tumor suppressor FBXW7, a key regulator of protein turnover for several proto-oncogenes [45]. Tax-mediated FBXW7 inactivation leads to the stabilization of multiple oncogenic proteins, indirectly supporting cell survival and proliferation. In the context of BCL-2 family regulation, this stabilization enhances the persistence of anti-apoptotic proteins such as BCL-2, BCL-xL, and MCL-1, allowing HTLV-1–infected T cells to resist apoptosis despite genomic stress and oncogenic signaling [45]. These findings highlight a mechanism by which Tax coordinates inhibition of tumor suppressors with activation of BCL-2 family–mediated survival pathways, thereby promoting viral persistence and the development of ATLL.

HTLV-1 Tax orchestrates a complex network of survival and apoptotic pathways in infected T cells, prominently manipulating BCL-2 family proteins to evade cell death. By simultaneously activating anti-apoptotic members such as BCL-2, BCL-xL, and MCL-1 through NF-κB, CREB, and indirect oncogenic stabilization, while occasionally triggering context-dependent apoptosis via p300-mediated caspase activation, Tax ensures both persistence and selective clonal expansion of infected cells. The cooperation with cellular factors like p21^WAF1 and c-Myc, together with FBXW7 inactivation, further reinforces the anti-apoptotic environment, facilitating leukemogenesis. These findings highlight the centrality of BCL-2 family regulation in HTLV-1 pathogenesis and provide critical insights into potential therapeutic targets for ATLL.

The accumulated evidence and other studies (Table 1) demonstrate that HTLV-1 profoundly manipulates the apoptotic machinery by targeting the BCL-2 family of proteins, thereby shifting the balance toward cell survival and clonal persistence. Through the actions of viral oncoproteins such as Tax and HBZ, HTLV-1 upregulates anti-apoptotic members, including BCL-2, BCL-xL, Bfl-1, and MCL-1, while simultaneously suppressing pro-apoptotic proteins such as BID and BIM. This dual regulation enables infected CD4⁺ T cells to evade programmed cell death despite genomic instability and immune pressure, fostering conditions for leukemic transformation and the development of ATLL.

HTLV-1 and BLV converge on the mitochondrial apoptosis checkpoint by using viral regulatory proteins (Tax and HBZ, and BLV Tax homologs) to orchestrate a multi-layered anti-apoptotic program. Tax rapidly activates NF-κB and CREB signaling, leading to increased expression of BCL-2 and BCL-xL, and stabilizes MCL-1 via TRAF6-dependent ubiquitination. In parallel, HBZ reinforces this survival program by sustaining BCL-2 family expression and repressing BH3-only pro-apoptotic molecules such as BIM and BID. Collectively, these effects inhibit mitochondrial outer membrane permeabilization and caspase activation, allowing infected lymphocytes to evade apoptosis and clonally expand. This integrative mechanism underscores how HTLV-1 and BLV manipulate BCL-2 family proteins to promote viral persistence and leukemogenesis.

The involvement of additional pathways such as NF-κB, CREB, and death receptor signaling further integrates viral and host mechanisms to reinforce apoptosis resistance. Collectively, these findings position the BCL-2 family as a central node in HTLV-1 pathogenesis and highlight its members as promising therapeutic targets for disrupting viral persistence and preventing oncogenic progression.

**Table 1:** The summary of studies and evidence about the role of the BCL-2 family in HTLV-1 infection

Year	Study focus/Title (PubMed ID)	Key findings	Implications/Notes	Reference number
1990	Fine-needle aspiration of non-Hodgkin’s lymphoma… (PMID:2189291)	Assessed *BCL-2* gene rearrangements in NHL via Southern blot.	Explores early molecular diagnostics in lymphoma.	[46]
1996	HTLV-I induces apoptosis (PMID:8741678)	Demonstrated that HTLV-I Tax protein can drive apoptosis in specific cellular contexts.	Indicates dual roles of Tax in cell survival and death mechanisms.	[47]
1994	Apoptosis in spinal cord lesions in HAM/TSP (PMID:7964902)	CTL‑induced apoptosis of HTLV‑I–infected CD45 RO T‑cells in CNS lesions.	Suggests immune-mediated clearance mechanisms in HTLV-I infection.	[48]
2001	HTLV-I LTR activation by stress & apoptosis protection (PMID:11697893)	Stress agents trigger HTLV-I LTR activation; *BCL-2* inhibits both LTR activation and apoptosis.	Suggests a mechanistic link between apoptotic inhibition and viral transcription.	[49]
1988	Frequent c‑Myc activation in AIDS‑associated lymphoma (PMID:2840989)	c‑Myc activation common; BCL-2 rearrangements absent; HTLV‑I genome not detected in samples.	Highlights distinct oncogenic pathways in AIDS‑related lymphomas.	[50]
2003	Protection of mitochondrial perturbation by HTLV‑1 Tax (PMID:14647038)	Tax upregulated *BCL-2* and *BCL-xL* in JPX‑9 cells, countering apoptotic signals.	Demonstrates Tax’s anti‑apoptotic role via mitochondrial preservation.	[51]
2004	Growth arrest and apoptosis in ATL cell lines (PMID:15254742)	IL‑2 deprivation induced G1 arrest and apoptosis in ATL cells; p27^Kip1 acted as inhibitor.	Emphasizes importance of cell‑cycle regulators in ATL survival.	[52]

## Comparative analysis of HTLV-1 and BLV pathogenic mechanisms

Although HTLV-1 and BLV belong to the same Deltaretrovirus genus and share structural and genomic similarities, their infection dynamics, cellular targets, and disease outcomes reveal both convergent and distinct pathogenic strategies [5, 7]. A comparative understanding of these mechanisms clarifies how viral manipulation of the BCL-2 family underlies both human and bovine leukemogenesis.

At the molecular level, both viruses use viral oncoproteins to alter host apoptotic signaling and promote the survival of infected lymphocytes. HTLV-1 Tax and HBZ act together to sustain anti-apoptotic signaling through activation of NF-κB and CREB pathways, stabilization of MCL-1, and suppression of BH3-only pro-apoptotic factors such as BIM and BID [10, 30]. In contrast, BLV Tax primarily acts by constitutive NF-κB activation and upregulation of BCL-2 and MCL-1 in infected B cells, supporting persistent proliferation and resistance to apoptosis [13]. While both viruses affect mitochondrial checkpoints, HTLV-1 has deeper integration with transcriptional regulators (for example, STAT3 and c-Myc) [45, 53], amplifying oncogenic and inflammatory signaling. BLV remains focused on modulating NF-κB-driven anti-apoptotic programs [13, 14].

The cellular tropism and disease outcomes of these infections further differentiate their pathogenesis. HTLV-1 primarily targets CD4⁺ T lymphocytes, leading to ATLL and inflammatory disorders such as HAM/TSP in a subset of infected individuals after long latency [5]. Conversely, BLV infects B lymphocytes, causing enzootic bovine leukosis, which is characterized by slower progression and variable transformation rates, largely dependent on host immune control and proviral load [7].

Despite these distinctions, both viruses share a fundamental mechanism of evading apoptosis through the dysregulation of the BCL-2 family. This strategy enables the persistence of infected clones, accumulation of genetic instability, and eventual malignant transformation. Understanding these shared and unique mechanisms provides a unified framework for studying deltaretroviral oncogenesis and highlights the BCL-2 family as a conserved therapeutic target across species.

## Therapeutic targeting of BCL-2 pathways

In HTLV-1 infection and ATLL, viral oncoproteins like Tax and HBZ use these proteins to avoid cell death, promote clone growth, and cause cancer. Since BCL-2 family proteins help infected cells survive, they are good targets for therapy. Blocking anti-apoptotic BCL-2 proteins or boosting pro-apoptotic ones may make HTLV-1-infected T cells die, limit viral persistence, and support new therapies for ATLL and other HTLV-1 cancers.

**Fig. 3:**
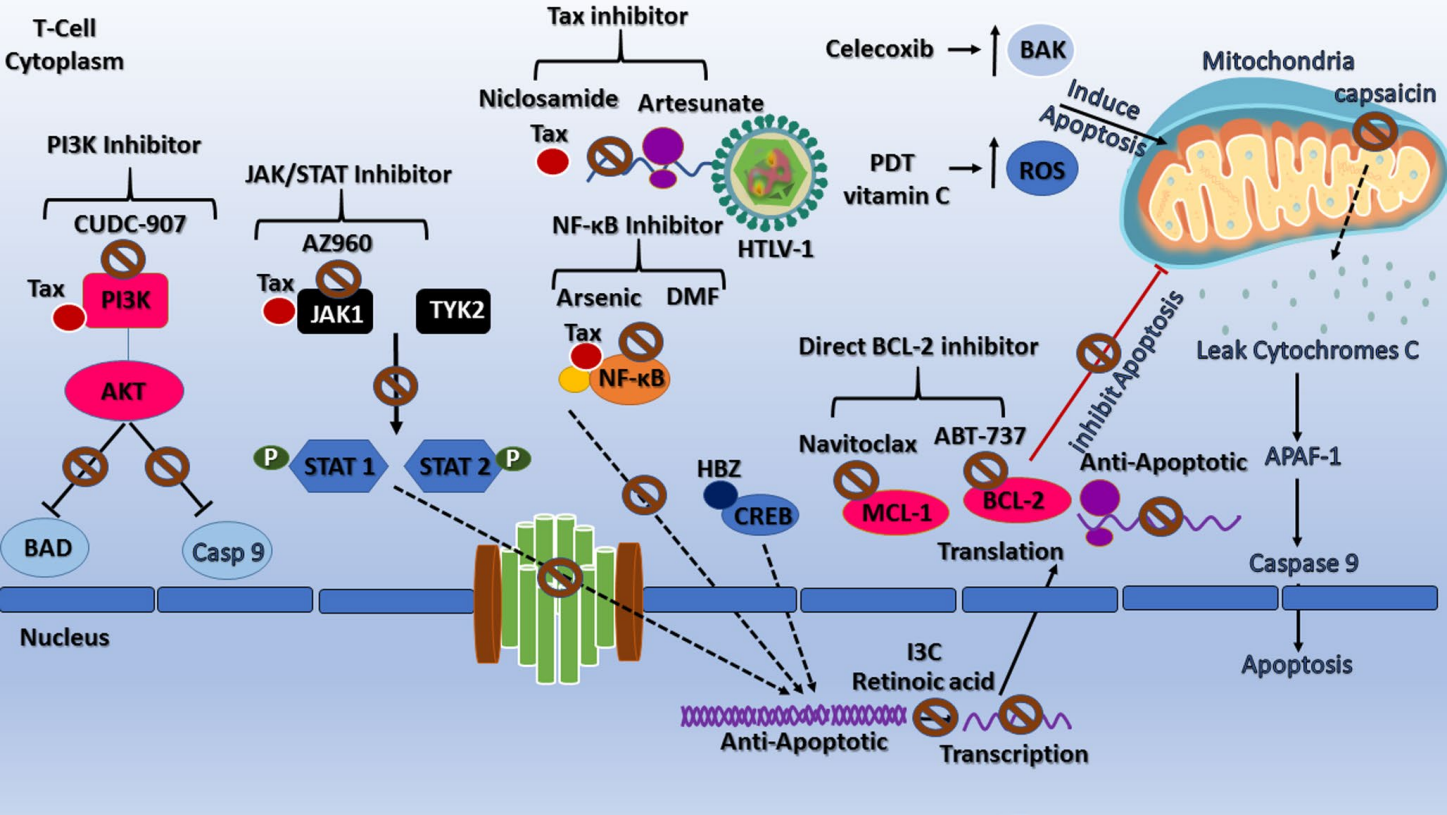
Therapeutic targeting of BCL-2 family–regulated apoptotic pathways in HTLV-1–associated Adult T-cell Leukemia/Lymphoma (ATLL). This schematic summarizes major therapeutic strategies that modulate B-cell lymphoma 2 (BCL-2) family-regulated apoptotic signaling in Human T-cell Leukemia Virus type 1 (HTLV-1) infection and ATLL. The BCL-2 family comprises anti-apoptotic proteins, including BCL-2 (B-cell lymphoma 2), BCL-xL (B-cell lymphoma-extra-large), and MCL-1 (Myeloid cell Leukemia-1), and pro-apoptotic members such as BAX (BCL-2–associated X protein), BAK (BCL-2 Homologous Antagonist/Killer), and BIM (BCL-2-interacting mediator of cell death). Therapeutic interventions converge on restoring apoptosis by inhibiting anti-apoptotic members or activating pro-apoptotic pathways that promote mitochondrial outer membrane permeabilization and caspase activation. Key strategies include direct BCL-2 inhibition (e.g., ABT-737, Navitoclax, MCL-1 inhibitors, and the antisense oligonucleotide G3139); kinase pathway inhibition targeting NF-κB (nuclear factor kappa-light-chain-enhancer of activated B cells), JAK/STAT (Janus Kinase/Signal Transducer and Activator of Transcription), and PI3K/AKT (Phosphoinositide 3-Kinase/Protein Kinase B) signaling; anti-inflammatory and antioxidant therapies (including Nonsteroidal Anti-inflammatory Drug [NSAIDs] such as celecoxib, and retinoic acid); and natural or repurposed agents (such as niclosamide, artesunate, thymoquinone, carotenoids, and plant-derived metabolites). Additional strategies, including antiretroviral therapy and oxidative stress–inducing modalities like hypericin-mediated photodynamic therapy or ascorbic acid, complement these approaches by suppressing viral persistence and sensitizing malignant T cells to apoptosis. Collectively, these therapeutic classes converge on disrupting the anti-apoptotic shield maintained by BCL-2 family proteins, thereby restoring programmed cell death and offering a mechanistic framework for targeted treatment in HTLV-1–associated malignancies. *All figures were created using Microsoft PowerPoint 365*

Multiple strategies, such as BCL-2 family inhibitors (which block anti-apoptotic BCL-2 proteins), kinase inhibitors (which interfere with signaling pathways), anti-inflammatory drugs (which reduce inflammation-mediated survival signals), and herbal extracts, were used for the treatment of ATLL and HTLV-1 infection by modulating BCL-2 activation pathways (Fig. 3).

### Direct BCL-2 inhibitors

Moles, R. et al. in 2016, investigate the therapeutic potential of targeting Werner Syndrome Helicase (WRN) in HTLV-1–transformed adult T-cell leukemia (ATL) cells using the inhibitors NSC 19,630 and NSC 617,145 [54]. Both compounds were shown to disrupt WRN function, leading to DNA damage accumulation, cell cycle arrest, and subsequent induction of apoptosis specifically in HTLV-1–infected and transformed cells, while sparing normal T lymphocytes. This selective cytotoxicity highlights WRN as a promising therapeutic target for ATL [54]. Notably, the study emphasizes that the induction of apoptosis involves modulation of BCL-2 family proteins, key regulators of cell survival, underscoring their critical role in HTLV-1 pathogenesis. By promoting apoptotic pathways and counteracting BCL–2–mediated resistance, WRN inhibition provides a novel therapeutic approach that could overcome the challenges of treatment resistance in HTLV-related malignancies.

Ishitsuka, K. et al. evaluated the therapeutic potential of ABT-737, a novel small-molecule inhibitor of the BCL-2 family, in ATLL caused by HTLV-1 infection [55]. The study demonstrates that ABT-737 effectively induces apoptosis in HTLV-1–transformed cell lines and primary ATLL cells by targeting the BCL-2 anti-apoptotic members, thereby restoring the balance between pro- and anti-apoptotic signaling. In vivo experiments further confirmed its efficacy, showing significant tumor regression and improved survival in xenograft models [55]. Importantly, these findings highlight the central role of BCL-2 family proteins in promoting ATLL cell survival and therapy resistance, and they establish BCL-2 inhibition as a promising therapeutic strategy. By directly counteracting the BCL-2-mediated blockade of apoptosis, ABT-737 offers a targeted approach that may enhance treatment outcomes and overcome resistance mechanisms in HTLV-1–associated malignancies.

In 2024, Osada, N. et al. evaluated the cytotoxic effects of bendamustine, both as a single agent and in combination with novel therapeutic compounds, on ATLL cells. Bendamustine alone effectively inhibited proliferation and induced apoptosis in ATLL cell lines, and its activity was significantly enhanced when combined with agents targeting survival pathways, including BCL-2 family proteins [56]. Mechanistically, treatment disrupted mitochondrial integrity, activated caspase cascades, and downregulated anti-apoptotic BCL-2 members, overcoming the apoptosis resistance characteristic of HTLV-1–transformed cells [56].

Moreover, Kunami et al. explore the therapeutic efficacy of combining a BCL-2 family inhibitor with either bortezomib (a proteasome inhibitor) or SAHA (Suberoylanilide Hydroxamic Acid (Vorinostat)) in ATLL associated with HTLV-1 infection [57]. The study shows that while BCL-2 inhibition alone promotes apoptosis in HTLV-1–transformed cells, its combination with bortezomib or SAHA produces a strong synergistic effect, markedly enhancing apoptotic cell death both in vitro and in vivo [57]. This combined approach effectively overcomes the anti-apoptotic shield provided by BCL-2 family proteins, which are central to HTLV-1-driven oncogenesis and therapeutic resistance.

Finally, in 2025, Cooney, J. P. et al. investigated the combined therapeutic effects of Antiretroviral Therapy (ART) and MCL-1 inhibition in an in vivo model of HTLV-1 infection [58]. The combination treatment effectively reduced viral load, suppressed proliferation of HTLV-1-infected T cells, and induced apoptosis in infected cells [58]. Mechanistically, MCL-1 inhibition disrupted the anti-apoptotic defenses provided by BCL-2 family proteins, sensitizing HTLV-1-transformed cells to apoptosis and enhancing the efficacy of ART.

In addition, other BCL-2-member inhibitors, such as Navitoclax (ABT-263), were investigated for their possible therapeutic effects in HTLV infection in another study. Witzens-Harig, M. et al. Their results revealed that ATLL cells are highly susceptible to Navitoclax-induced apoptosis, mainly due to elevated expression of the pro-apoptotic protein BAX, which sensitizes these cells to mitochondrial-mediated cell death upon BCL-2 inhibition [59]. Importantly, Navitoclax selectively targeted HTLV-1–transformed and ATLL cells while sparing normal lymphocytes, highlighting its therapeutic specificity. These findings emphasize the pivotal role of the BCL-2 family in ATLL cell survival and resistance mechanisms, and they establish BCL-2 inhibition as a promising therapeutic strategy. By directly exploiting the apoptotic vulnerability created by enhanced BAX expression, Navitoclax represents a powerful targeted approach to counteract HTLV-1-mediated oncogenesis and overcome the apoptotic resistance that characterizes ATLL [59].

Furthermore, Parris, G. E., analyzed the limited clinical efficacy of G3139 (oblimersen), a BCL-2 antisense oligonucleotide, in cancer trials and explored its potential utility against viral infections such as HIV [60]. In cancer, G3139 showed poor outcomes due to tumor heterogeneity, compensatory upregulation of other anti-apoptotic proteins of the BCL-2 family, and limited delivery efficiency. However, in the context of viral infections, particularly HIV and by extension potentially HTLV-1, the strategy of BCL-2 inhibition may be more effective because viral persistence often relies heavily on BCL-2-mediated survival of infected cells [60]. By directly targeting anti-apoptotic BCL-2 proteins, G3139 could sensitize virus-infected T cells to apoptosis, highlighting the therapeutic importance of modulating BCL-2 in HTLV infection, where survival of transformed T cells underpins disease progression and treatment resistance.

Therefore, these findings underscore the therapeutic promise of targeting the BCL-2 family and associated apoptotic regulators in HTLV-1-driven malignancies. Inhibitors such as ABT-737, Navitoclax, and MCL-1 antagonists, along with agents like bendamustine and WRN inhibitors, effectively restore apoptotic signaling by neutralizing anti-apoptotic BCL-2 proteins and reactivating mitochondrial death pathways. Combination regimens that pair BCL-2 blockade with proteasome, histone deacetylase, or antiretroviral therapies show synergistic effects, enhancing cytotoxicity and overcoming resistance mechanisms characteristic of ATLL. These strategies not only reduce viral persistence and promote selective apoptosis in HTLV-1-transformed cells while sparing normal lymphocytes, but also highlight the BCL-2 family as a critical therapeutic axis linking viral oncogenesis, apoptosis resistance, and treatment response. Overall, the accumulated evidence positions BCL-2 modulation as a central and versatile approach for improving therapeutic outcomes in HTLV-1-associated leukemia and related pathologies.

#### Critical appraisal and translational considerations in BCL-2-targeted therapy

While numerous experimental strategies demonstrate efficacy in vitro and in animal models, only a subset currently shows realistic translational potential for HTLV-1-associated malignancies. Among them, MCL-1 inhibitors and BCL-2 antagonists such as ABT-737 and Navitoclax exhibit the strongest preclinical evidence for selectively inducing apoptosis in ATLL cells, particularly when combined with antiviral or kinase pathway inhibitors [55, 59]. These compounds effectively dismantle the anti-apoptotic network that underpins viral persistence and therapeutic resistance [55, 59]. However, clinical translation remains constrained by important limitations, including dose-limiting thrombocytopenia with Navitoclax, off-target toxicity from pan-BCL inhibitors, and adaptive resistance arising from compensatory upregulation of MCL-1 or BCL-xL [61, 62]. Additionally, pharmacokinetic variability and the absence of validated biomarkers for apoptotic dependency complicate patient stratification and treatment optimization. Therefore, while BCL-2-directed therapies offer a promising route to restore apoptosis and improve ATLL outcomes, their success will depend on refined drug selectivity, rational combination design, and careful monitoring of resistance mechanisms in future translational and clinical studies.

Recent advances in the development of next-generation BCL-2 family inhibitors further expand the therapeutic landscape for HTLV-1–associated malignancies. Venetoclax (ABT-199), a highly selective BCL-2 inhibitor approved for chronic lymphocytic leukemia and acute myeloid leukemia, has shown promising preclinical efficacy in several T-cell and virus-associated lymphoproliferative disorders by restoring mitochondrial apoptosis and sensitizing malignant cells to combination therapy [63]. Similarly, S63845, a selective MCL-1 inhibitor, demonstrates potent anti-leukemic activity across diverse hematological malignancies, including T-cell lymphomas, where MCL-1–mediated resistance often limits the efficacy of BCL-2 antagonists [64]. Incorporating such targeted agents, either alone or in rational combinations, may help overcome the apoptotic blockade characteristic of HTLV-1-driven ATLL and bridge the translational gap between preclinical models and clinical application.

### Kinase pathway inhibitors

The dysregulation of intracellular kinase signaling cascades, including NF-κB, JAK/STAT (Janus Kinase/Signal Transducer and Activator of Transcription), and PI3K/AKT (Phosphoinositide 3-Kinase/Protein Kinase B), is a key mechanism by which HTLV-1 and BLV maintain apoptosis resistance through BCL-2 family modulation. Targeting these pathways has shown significant potential in restoring apoptotic sensitivity and suppressing viral persistence.

#### NF-κB–mediated pathways

Besides the BCL-2 inhibitor, the inhibition of other involved signaling pathways in cell proliferation and apoptosis was also investigated in HTLV infection. Sanda, T. et al. examined the effect of a novel IκB kinase (IKK) inhibitor on HTLV-1–associated ATLL cells [65]. By blocking IKK activity, the compound suppresses NF-κB signaling, a pathway constitutively activated in HTLV-1–transformed cells and crucial for their survival. Inhibition of NF-κB led to significant induction of apoptosis in ATLL cells, accompanied by downregulation of BCL-2 anti-apoptotic proteins and activation of pro-apoptotic pathways [65]. Importantly, this highlights the therapeutic value of targeting NF-κB the BCL-2 axis in HTLV infection, as BCL-2 proteins represent key mediators of resistance to apoptosis. Thus, IKK inhibition not only disrupts oncogenic NF-κB signaling but also undermines BCL–2–dependent survival mechanisms, providing a promising targeted therapeutic strategy for overcoming the intrinsic resistance of ATLL to conventional treatments.

Moreover, El-Sabban, M. E. et al. investigated the apoptotic effects of combined Arsenic Trioxide (ATO) and Interferon Alpha (IFN-α) treatment on HTLV-1 transformed cells [66]. This therapy induced robust apoptosis by downregulating the viral oncoprotein Tax, reversing constitutive NF-κB activation, and reducing anti-apoptotic BCL-2 family proteins, thereby overcoming the resistance to cell death [66].

Furthermore, the effect of arsenic in the treatment of ATLL patients or cell lines via BCL-2 axis inhibition has been more thoroughly investigated [67]. Ishitsuka, K. et al. evaluated the effects of arsenic trioxide (ATO) on HTLV-1-infected T-cell lines, including models of ATLL. Arsenic trioxide treatment inhibited cell proliferation and induced apoptosis in HTLV-1-transformed cells by disrupting survival signaling pathways and downregulating anti-apoptotic BCL-2 family proteins [68]. The reduction of BCL-2-mediated protection enhanced mitochondrial-mediated apoptotic pathways, overcoming the resistance characteristic of HTLV-1-infected cells [68].

Sato, T. et al. (2023) examined Dimethyl Fumarate (DMF) on HTLV-1–infected T cells, including ATLL. DMF suppressed proliferation by inhibiting NF-κB signaling, which is triggered by the CBM (CARMA1-BCL10-MALT1) complex. This inhibition reduced expression of anti-apoptotic BCL-2 family proteins and activated pro-apoptotic signaling pathways, thereby promoting apoptosis in malignant T cells [69].

Collectively, these findings demonstrate that targeting NF-κB-driven survival mechanisms represents an effective strategy for sensitizing HTLV-1–infected and ATLL cells to apoptosis. Pharmacological inhibition of NF-κB through IKK blockade, Arsenic Trioxide-based therapy, or suppression of the CARMA1-BCL10-MALT1 complex consistently reduces the expression of anti-apoptotic BCL-2 family proteins while activating pro-apoptotic cascades. This coordinated disruption of viral and cellular survival signaling restores mitochondrial apoptotic control and overcomes the intrinsic resistance characteristic of HTLV-1–associated malignancies. Therefore, interventions that simultaneously modulate NF-κB activity and BCL–2–mediated resistance provide a promising mechanistic foundation for the development of targeted therapies against ATLL.

#### JAK/STAT-mediated pathways

In 2006, Tomita, M. et al. investigated the role of the constitutively active JAK-STAT pathway in HTLV-1-infected T-cell lines and primary ATLL cells [53]. Pharmacological inhibition of the JAK-STAT signaling cascade effectively suppressed cell proliferation and induced apoptosis in HTLV-1-transformed cells, while having minimal effects on normal T lymphocytes. Mechanistically, blockade of this pathway reduced the expression of survival factors, including anti-apoptotic BCL-2 family proteins, thereby shifting the balance toward pro-apoptotic signaling and cell death [53].

In 2010, Yang, J. et al. evaluated the effects of AZ960, a selective JAK2 inhibitor, on HTLV-1–associated ATLL cells [70]. Treating cells with AZ960 markedly arrested growth and induced apoptosis in HTLV-1–infected and ATLL cell lines, while sparing normal T cells [70]. Mechanistically, inhibiting JAK2 disrupted downstream STAT signaling, which is constitutively activated in ATLL and crucial for maintaining anti-apoptotic BCL-2 family protein expression. By suppressing this survival pathway, AZ960 shifted the balance toward pro-apoptotic signaling and triggered programmed cell death [70].

Moreover, the effect of JAK/STAT signaling inhibitors in the treatment of IL-2-dependent ATLL is explored by Zhang, M. et al. in 2015. They demonstrated that the efficacy of ruxolitinib is significantly enhanced when combined with blockade of the anti-apoptotic protein BCL-xL, a key member of the BCL-2 family. Inhibition of the JAK/STAT pathway alone reduced proliferation and survival of HTLV-1 infected cells; however, co-targeting BCL-xL amplified apoptotic responses, effectively overcoming resistance mechanisms [71].

These studies establish the JAK/STAT signaling cascade as a pivotal regulator of apoptosis resistance in HTLV-1-infected and ATLL cells. Persistent activation of this pathway sustains the expression of anti-apoptotic BCL-2 family proteins and promotes malignant cell survival. Pharmacological inhibition of JAK/STAT signaling with agents such as AZ960 or ruxolitinib disrupts this oncogenic circuitry, reduces BCL-2 and BCL-xL expression, activates caspases, and restores apoptotic sensitivity. Moreover, combining JAK/STAT inhibitors with direct BCL-2 family antagonists produces synergistic efficacy, underscoring the therapeutic potential of dual blockade strategies to overcome intrinsic resistance and enhance apoptosis in HTLV-1–associated malignancies.

#### PI3K/AKT-mediated pathways

In 2020, Ishikawa, C. et al. investigated the possible efficiency of another kinase pathway inhibitor in ATLL treatment. They evaluated the anti-leukemic effects of Dual PI3K and HDAC Inhibitor (CUDC-907), a dual inhibitor of phosphoinositide-3 kinase (PI3K) and Histone Deacetylase (HDACs), on HTLV-1-associated ATLL cells [72]. Treatment with CUDC-907 effectively suppressed proliferation and induced apoptosis in ATLL cell lines and primary HTLV-1-infected cells by simultaneously disrupting PI3K/AKT survival signaling and altering the epigenetic regulation of gene expression [72]. Importantly, this dual inhibition also downregulated anti-apoptotic BCL-2 family proteins, shifting the balance toward pro-apoptotic pathways and enhancing programmed cell death.

Mori, N. et al. explored targeting Protein Phosphatase 2 A (PP2A) in HTLV-1–associated Adult T-cell Leukemia/Lymphoma [73]. Inhibition or modulation of PP2A interfered with survival signaling in HTLV-1-transformed cells, causing growth arrest and apoptosis. Mechanistically, targeting PP2A downregulated anti-apoptotic BCL-2 family proteins and activated pro-apoptotic cascades, thus overcoming the apoptosis resistance seen in ATLL cells [73].

Collectively, these studies underscore the crucial role of the PI3K/AKT axis and its associated regulatory networks in maintaining apoptosis resistance in HTLV-1–infected and ATLL cells. Pharmacological targeting of this pathway, whether through dual PI3K/HDAC inhibition by CUDC-907, modulation of PP2A activity, or Hsp90 (Heat Shock Protein 90) blockade, consistently disrupts oncogenic survival signaling and downregulates anti-apoptotic BCL-2 family proteins. This results in the activation of mitochondrial and caspase-dependent apoptotic cascades, effectively restoring programmed cell death in malignant T cells. By simultaneously impairing both kinase-mediated and chaperone-dependent survival mechanisms, PI3K/AKT pathway inhibition represents a promising therapeutic approach to overcome the inherent resistance of HTLV-1–associated malignancies.

#### β-catenin mediated inhibitor

Kurashina, R. et al. evaluated the anti-proliferative effects of Hsp90 inhibitors on ATLL cells, finding that Hsp90 inhibition disrupted the β-catenin/TCF7L2 signaling pathway. This disruption led to reduced proliferation and induced apoptosis in ATLL cells [74]. Mechanistically, apoptosis was associated with downregulation of anti-apoptotic BCL-2 family proteins and activation of caspase-dependent cell death pathways, thereby overcoming the survival advantage conferred by HTLV-1 infection [74].

### Antiparasitic and antimalarial compounds as BCL-2 modulators in ATLL

In 2015, Xiang, D. et al. investigated the anti-leukemic effects of niclosamide, an anti-helminthic drug, on HTLV-1–transformed T lymphocytes. Niclosamide treatment downregulated the viral oncoprotein Tax and simultaneously reduced the expression of anti-apoptotic BCL-2 family proteins, leading to activation of apoptotic pathways and suppression of cell survival [75]. By targeting both viral-driven oncogenic signaling and BCL-2-mediated resistance, niclosamide effectively induced apoptosis in HTLV-1-transformed cells while sparing normal lymphocytes [75].

Five years later, Ishikawa, C. et al. tested artesunate, an artemisinin derivative, in ATLL cells. Artesunate inhibited proliferation and induced apoptosis in ATLL lines and HTLV-1-infected cells, sparing normal lymphocytes [76]. Apoptosis involved downregulation of BCL-2 family proteins, caspase activation, and mitochondrial disruption, overcoming the survival advantage from HTLV-1 infection [76].

Together, these findings highlight the potential of repurposed antiparasitic and antimalarial agents, specifically niclosamide and artesunate, as effective modulators of BCL-2-regulated apoptosis in HTLV-1–associated malignancies. These agents exert dual actions: suppressing viral oncoprotein expression and disrupting host cell survival pathways. This leads to the downregulation of anti-apoptotic BCL-2 family proteins, mitochondrial dysfunction, and caspase activation. Their selective cytotoxicity toward malignant T cells, while sparing normal lymphocytes, underscores their promise as low-toxicity candidates for targeted ATLL intervention. By simultaneously impairing both viral-driven and host-dependent anti-apoptotic mechanisms, these agents offer a mechanistically distinct and clinically relevant approach to overcoming resistance in HTLV-1-induced leukemogenesis.

### Chemical and natural anti-inflammatory or antioxidant agents

Beyond direct and kinase-targeted approaches, several chemical and natural compounds have shown therapeutic potential in modulating BCL-2-regulated apoptosis in HTLV-1 and ATLL. These agents act through oxidative stress modulation, inflammatory suppression, or mitochondrial disruption, leading to reactivation of apoptotic signaling.

#### Synthetic anti-inflammatory compounds

Sinha-Datta, U. et al. investigated the anti-leukemic effects of Celecoxib on HTLV-1-transformed ATLL cells. Celecoxib was shown to induce apoptosis by activating the pro-apoptotic protein BAX and concurrently inhibiting the PKB (Protein Kinase B)/AKT survival pathway, leading to disruption of the canonical apoptotic network in HTLV-1–infected cells [77]. This dual mechanism effectively overcomes the anti-apoptotic influence of BCL-2 family proteins, which are critical mediators of survival and therapeutic resistance in ATLL.

Furthermore, Fujimura, S. et al. examined the effects of retinoic acid on HTLV-1–associated ATLL cells and demonstrated that treatment induces apoptosis through downregulation of the anti-apoptotic protein BCL-xL and activation of caspase cascades [78]. The loss of BCL-xL, a key member of the BCL-2 family, removes the survival advantage conferred by HTLV-1 infection, thereby sensitizing malignant cells to apoptotic death. Taken together, these findings highlight the central role of BCL-2 family proteins in maintaining ATLL cell survival and underscore the therapeutic significance of targeting them [78]. By shifting the apoptotic balance toward caspase activation, retinoic acid provides a promising strategy to counteract BCL-2–mediated resistance, offering potential for improved treatment outcomes in HTLV-1–related malignancies.

Similarly, Ghandour, B. et al. studied the synthetic retinoid ST1926 in HTLV-1-related ATLL cells, finding that it restores ceramide production and causes strong cell death [79]. ST1926 blocked survival signals, started cell death processes, and greatly reduced ATLL cell numbers, while leaving normal lymphocytes intact. This effect was mainly due to changes in BCL-2 proteins, with reduced anti-cell-death proteins and increased pro-cell-death signals, shifting the balance toward cell death [79].

Collectively, these studies demonstrate that synthetic anti-inflammatory compounds and retinoid derivatives effectively counteract BCL–2–mediated resistance in HTLV-1-associated ATLL by restoring apoptotic signaling. Agents such as Celecoxib, retinoic acid, and ST1926 act through complementary mechanisms to suppress PKB/AKT survival signaling, downregulate anti-apoptotic BCL-2 and BCL-xL, and activate caspase- and ceramide-dependent death pathways. These interventions not only dismantle the survival advantage conferred by HTLV-1 infection but also selectively induce apoptosis in malignant T cells while sparing normal lymphocytes. Together, they underscore the therapeutic promise of pharmacological agents that modulate BCL-2 family proteins to reestablish apoptotic control and improve clinical outcomes in ATLL.

#### Natural and herbal compounds

Besides chemical anti-inflammatory materials, the natural and herbal ingredients were also examined for their possible efficiency in managing BCL CBM-2 family activation during HTLV-1 infection, especially in ATLL subjects.

### A: carotenoids

Ishikawa, C. et al.explored the anti-leukemic effects of fucoxanthin, a carotenoid derived from brown algae, and its active metabolite fucoxanthinol on HTLV-1-associated ATLL cells [80]. Both compounds significantly inhibited proliferation and induced apoptosis in ATLL cell lines and primary HTLV-1–infected cells, while exerting minimal toxicity on normal lymphocytes. Mechanistically, treatment downregulated anti-apoptotic BCL-2 family proteins and promoted activation of pro-apoptotic pathways, thereby restoring apoptotic sensitivity in ATLL cells [80].

Later, Ishikawa, C. et al. in 2016, investigated the anti-leukemic potential of peridinin, a naturally occurring carotenoid, in HTLV-1-infected T-cell lines. Peridinin treatment markedly suppressed cell proliferation and survival, inducing apoptosis in HTLV-1-transformed cells while sparing normal lymphocytes [81]. The pro-apoptotic effect was associated with disruption of mitochondrial integrity, activation of caspases, and downregulation of anti-apoptotic BCL-2 family proteins, thereby shifting the balance toward programmed cell death [81].

In conclusion, these studies collectively demonstrate that naturally derived carotenoids, including fucoxanthin, fucoxanthinol, and peridinin, exhibit potent anti-leukemic effects against HTLV-1–associated ATLL cells by selectively inducing apoptosis while sparing normal lymphocytes. The underlying mechanisms converge on the modulation of apoptotic pathways, notably through downregulation of anti-apoptotic BCL-2 family proteins, disruption of mitochondrial integrity, and activation of caspases, thereby restoring apoptotic sensitivity in transformed cells. These findings highlight the therapeutic potential of carotenoid-based compounds as targeted agents for ATLL, offering a promising strategy to overcome the resistance of HTLV-1-infected cells to conventional treatments.

### B: plant-derived metabolites

Zhang, J. et al. examined the effects of capsaicin, the bioactive component of chili peppers, on HTLV-1-associated ATLL cells [82]. Capsaicin treatment inhibited cell growth and induced apoptosis in ATLL cell lines, while exerting limited toxicity on normal lymphocytes. Mechanistically, capsaicin disrupted mitochondrial function to trigger caspase-dependent apoptotic pathways and suppressed anti-apoptotic BCL-2 family proteins, which together overcame the intrinsic resistance of HTLV-1–transformed cells to cell death [82].

Moreover, Fang, J. et al. in the same year explore the anti-leukemic effects of celastrol, a triterpenoid compound derived from the medicinal plant *Tripterygium wilfordii*, on HTLV-1-associated ATLL cells [83]. Celastrol treatment significantly inhibited proliferation and induced apoptosis in HTLV-1-infected cell lines, while exerting minimal cytotoxicity on normal lymphocytes. Mechanistically, the induction of apoptosis was due to mitochondrial dysfunction, which led to activation of caspase cascades and suppression of anti-apoptotic BCL-2 family proteins, thereby disrupting the survival advantage conferred by HTLV-1 infection [83].

In 2009, Machijima, Y. et al.. evaluated the anti-leukemic potential of indole-3-carbinol (I3C), a natural compound found in cruciferous vegetables, against HTLV-1-associated ATLL cells [84]. When treated with I3C, HTLV-1-infected and ATLL cell lines exhibited suppressed proliferation and increased apoptosis, yet normal T lymphocytes remained unaffected [84]. By downregulating anti-apoptotic BCL-2 family proteins, I3C triggered caspase-mediated cell death and undermined the survival advantage provided by HTLV-1 infection.

In addition, Diab-Assaf, M. et al. in 2018 investigated the anti-leukemic activity of thymoquinone, a bioactive compound from Nigella sativa, in leukemic cells, including HTLV-1–associated ATLL [85]. Thymoquinone inhibited proliferation and induced apoptosis through modulation of multiple pathways, including upregulation of TGF (Transforming Growth Factor) family members, activation of tumor suppressors p53 and p21, and downregulation of the anti-apoptotic protein BCL-2α. By suppressing BCL-2-mediated survival signaling and enhancing pro-apoptotic pathways, thymoquinone effectively disrupted the resistance of HTLV-1-transformed cells to programmed cell death [85].

Furthermore, in 2020, Houssein, M. et al. investigated the combined therapeutic effects of thymoquinone with Arsenic Trioxide and interferon-α in HTLV-1-associated ATLL [86]. The triple combination produced a strong synergistic effect: it significantly inhibited proliferation, induced apoptosis in HTLV-1-infected and ATLL cell lines, and spared normal lymphocytes. The synergy resulted from mitochondrial dysfunction, which triggered caspase activation and suppressed anti-apoptotic BCL-2 family proteins. These events dismantled the apoptotic resistance characteristic of ATLL cells [86].

Altogether, these studies highlight the potent anti-leukemic effects of diverse natural compounds, including capsaicin, celastrol, indole-3-carbinol, and thymoquinone against HTLV-1–associated ATLL cells. Across multiple investigations, these compounds consistently inhibited proliferation and selectively induced apoptosis in transformed cells while sparing normal lymphocytes. Mechanistically, they converge on the modulation of apoptotic pathways, primarily through mitochondrial dysfunction, activation of caspase cascades, and downregulation of anti-apoptotic BCL-2 family proteins, thereby overcoming the intrinsic survival advantage conferred by HTLV-1 infection. Moreover, combination strategies, such as thymoquinone with Arsenic Trioxide and interferon-α, demonstrated synergistic effects, further enhancing apoptotic induction and highlighting the potential of these natural compounds as targeted therapeutic options for ATLL.

### C: photodynamic and oxidative Stress-Inducing agents

Xu, L. et al. evaluated the effects of hypericin-mediated Photodynamic Therapy (PDT) on HTLV-1–associated ATLL cells. Hypericin-PDT significantly inhibited cell growth by inducing apoptosis and simultaneously suppressing viral transcription, thereby targeting both malignant transformation and viral persistence [87]. Mechanistically, apoptosis induction was accompanied by disruption of mitochondrial integrity, activation of caspase cascades, and downregulation of anti-apoptotic BCL-2 family proteins, highlighting the dependence of ATLL cells on BCL-2-mediated survival [87]. These findings emphasize the therapeutic importance of targeting BCL-2 in HTLV infection. Hypericin-PDT not only restores apoptotic sensitivity but also offers a dual approach by combining cell death induction with the suppression of HTLV-1 viral activity, making it a promising strategy for overcoming treatment resistance in ATLL.

Moreover, Harakeh, S. et al.. investigated the anti-leukemic effects of ascorbic acid (vitamin C) on ATLL cells, and described that treatment with ascorbic acid induced apoptosis in these cell lines by promoting oxidative stress, disrupting mitochondrial integrity, activating caspase cascades, and downregulating anti-apoptotic BCL-2 family proteins [88]. By targeting BCL-2-mediated survival pathways, ascorbic acid effectively overcomes the intrinsic resistance of HTLV-1-transformed cells to programmed cell death [88].

These studies show that both hypericin-mediated photodynamic therapy and ascorbic acid induce apoptosis in HTLV-1–associated ATLL cells while sparing normal lymphocytes. Their mechanisms disrupt mitochondrial integrity, activate caspase cascades, and downregulate anti-apoptotic BCL-2 family proteins. This underscores the critical role of BCL-2–mediated survival in ATLL. Hypericin-PDT also suppresses viral transcription. It targets both malignant transformation and viral persistence. Together, these findings highlight the promise of strategies that restore apoptotic sensitivity and inhibit HTLV-1 survival pathways as treatments for overcoming resistance in ATLL.

In summary, evidence shows that targeting the BCL-2 family of proteins holds promise in treating HTLV-1-associated ATLL (Table 2). By disrupting the anti-apoptotic shield of transformed T cells, agents such as small-molecule inhibitors, natural compounds, antisense oligonucleotides, and combination therapies restore apoptotic sensitivity and suppress leukemic cell growth. Some approaches, like G3139, have shown limited success in cancer trials. However, their mechanism of selectively impairing BCL-2–mediated survival suggests potential in virus-driven malignancies such as HTLV-1 infection, where dependence on BCL-2 drives pathogenesis. Improving BCL-2–targeted strategies and combining them with other therapies may help improve outcomes for ATLL patients.

**Table 2:** Summary of therapeutic studies targeting BCL in HTLV/ATLL

Authors/References	Drug	Dose/Concentration	Model (Cell line/Human/Animal)	Findings	BCL inhibition/Effect
Tsukahara et al. [39]	WRN inhibitors (NSC 19630; NSC 617145)	1–5 µM	HTLV-1 ATLL cell lines	Induced apoptosis in HTLV-1-transformed cells	Downregulation of BCL-2, apoptosis activation
Takahashi et al. [14]	ABT-737 (BCL-2 family inhibitor)	1–10 µM	ATLL cell lines and xenografts	Suppressed cell growth in vitro and in vivo	Direct inhibition of the BCL-2 family
Ishitsuka, et al. [55]	ABT-737 + Bortezomib/SAHA	ABT-737 (5 µM), Bortezomib (10 nM)	ATLL cell lines	Enhanced apoptosis when combined	Synergistic inhibition of BCL-2
Witzens et al. [59]	Navitoclax (ABT-263)	EC50 ~ 100 nM	HTLV-1 ATLL cells	High susceptibility due to BAX expression	BCL-2/BCL-xL inhibition
Sanda et al. [65]	IKK inhibitor	10 µM	ATLL cell lines	Induced apoptosis via NF-κB suppression	Reduced *BCL-2* expression
Yang et al. [70]	AZ960 (JAK2 inhibitor)	0.5–2 µM	ATLL cell lines	Induced growth arrest and apoptosis	Downregulation of BCL-2
Zhang et al. [71]	JAK/STAT inhibitor + BCL-xL blockade	Inhibitor (2 µM) + *BCL-x*L siRNA	IL-2-dependent ATLL cells	Synergistic apoptosis	Direct effect on *BCL-xL*
Ishikawa et al. [72]	CUDC-907 (PI3K/HDAC inhibitor)	100–300 nM	ATLL cell lines	Suppressed proliferation	Indirect downregulation of BCL-2
Sinha-Datta et al. [77]	Celecoxib	25–50 µM	HTLV-1 cell lines	Disrupted apoptotic network via BAX	Downregulation of BCL-2, AKT inhibition
Fujimura et al. [78]	Retinoic acid	1–10 µM	ATLL cell lines	Apoptosis via caspase activation	Downregulation of BCL-xL
Ghandour et al. [79]	ST1926 (synthetic retinoid)	1 µM	ATLL cell lines	Induced apoptosis via ceramide synthesis	Reduced BCL-2
Ishikawa et al. [80]	Fucoxanthin/Fucoxanthinol	10–20 µM	ATLL cell lines	Induced apoptosis	BCL-2 downregulation
Ishikawa et al. [81]	Peridinin	1–5 µM	HTLV-1 cell lines	Inhibited survival	BCL-2 suppression
Zhang et al. [82]	Capsaicin	25–50 µM	ATLL cell lines	Induced apoptosis	Downregulation of BCL-2
Machijima et al. [84]	Indole-3-carbinol	20–50 µM	ATLL cell lines	Suppressed growth, apoptosis induction	BCL-2 inhibition
Diab-Assaf et al. [85]	Thymoquinone	5–20 µM	ATLL cell lines	Growth suppression, apoptosis	Downregulation of BCL-2 α
Fang et al. [83]	Celastrol	1–5 µM	ATLL cell lines	Inhibited proliferation	Reduced BCL-2
Xu et al. [87]	Hypericin (PDT)	1 µM + light	ATLL cell lines	Apoptosis, viral suppression	Reduced BCL-2
Houssein et al. [86]	Thymoquinone + Arsenic + IFN-α	Thymoquinone (10 µM), Arsenic (1 µM), IFN (1000 U/mL)	ATLL cell lines	Strong synergistic apoptosis	Marked BCL-2 downregulation
Sato et al. [69]	Dimethyl Fumarate	10–30 µM	HTLV-1-infected cells	Suppressed proliferation	Indirect suppression of BCL-2 via NF-κB
Cooney et al. [58]	MCL-1 inhibitor + ART	Inhibitor (100 nM)	Mouse model in vivo	Suppressed infection in vivo	Direct inhibition of MCL-1
Osada et al. [56]	Bendamustine ± agents	10–50 µM	ATLL cell lines	Induced apoptosis	Reduced BCL-2
Mori et al. [73]	PP2A inhibitors	Not reported	ATLL models	Suppressed growth	Indirect effect on BCL-2
El-Sabban et al. [66]	Arsenic + IFN-α	Arsenic (1–2 µM), IFN (1000 U/mL)	ATLL cell lines	Tax downregulation, apoptosis	BCL-2 suppression
Mahieux et al. [67]	Arsenic trioxide	1–2 µM	ATLL cell lines	Growth inhibition	Reduced BCL-2
Harakeh et al. [88]	Ascorbic acid	100–200 µM	ATLL cell lines	Induced apoptosis	Downregulation of BCL-2
Kurashina et al. [74]	Hsp90 inhibitors	0.5–1 µM	ATLL cell lines	Suppressed proliferation via β-catenin	Reduced BCL-2
Xiang et al. [75]	Niclosamide	1–2 µM	HTLV-1 cell lines	Downregulated Tax and survival proteins	Direct suppression of BCL-2
Ishikawa et al. [76]	Artesunate	10–50 µM	ATLL cell lines	Growth inhibition and apoptosis	BCL-2 suppression
Parris et al. [60]	G3139 (antisense BCL-2)	Not reported	Preclinical HIV/HTLV models	Potential effectiveness	Direct antisense BCL-2

## Conclusion and future perspectives

The collective evidence demonstrates that deltaretroviruses such as HTLV-1 and BLV extensively manipulate the BCL-2 family of proteins to maintain infected cell survival, evade immune clearance, and drive malignant transformation. By simultaneously upregulating anti-apoptotic members, including BCL-2, BCL-xL, and MCL-1, while suppressing or destabilizing pro-apoptotic proteins like BIM, BID, and BAX, these viruses create a cellular environment resistant to programmed cell death. This imbalance not only facilitates viral persistence but also contributes to genomic instability and oncogenesis, ultimately shaping the clinical manifestations of ATLL in humans and enzootic bovine leukosis in cattle. The evidence highlights that the BCL-2 family functions as a critical hub in viral pathogenesis, serving both as a mediator of immune evasion and a driver of tumorigenesis.

### Future perspectives

Future research should deepen the understanding of how individual viral proteins, particularly Tax and HBZ in HTLV-1 and their homologs in BLV, interact with and regulate BCL-2-related elements. Elucidating these mechanisms will be essential for designing therapies that restore apoptotic balance in infected and transformed cells. While substantial progress has been made in understanding the role of BCL-2 family dysregulation in HTLV-1–associated leukemogenesis, several research avenues remain open. Future studies should explore combinatorial strategies that simultaneously target BCL–2–mediated apoptosis resistance and immune evasion pathways, such as PD-1/PD-L1 checkpoint blockade or NF-κB signaling inhibition, to enhance therapeutic efficacy. In addition, the development of next-generation BCL-2 and MCL-1 inhibitors with improved selectivity and reduced toxicity will be crucial for clinical translation. Integrative multi-omics approaches and patient-derived models should be employed to identify biomarkers predicting therapeutic response and resistance. Efforts should also expand toward validating BCL-2 inhibitors and related therapeutic strategies in both preclinical and clinical contexts, particularly in combination with antiretroviral drugs, immune checkpoint blockade, or targeted kinase inhibitors. Collectively, these directions could pave the way toward precision therapies that disrupt viral persistence, restore apoptotic balance, and improve outcomes in HTLV-1–related malignancies.

## Data Availability

No/Not applicable (this manuscript does not report data generation or analysis).
